# Effects on Glycaemic Control by Continuous Intravenous Regular Insulin With or Without Subcutaneous Glargine Basal Insulin in Patients With Diabetes Following Coronary Artery Bypass Grafting Surgery

**DOI:** 10.7759/cureus.101357

**Published:** 2026-01-12

**Authors:** Md. Salahuddin Rahaman, Iram Shahazadi, Md. Faisal Nafis Majumder, Sumit Barua

**Affiliations:** 1 Cardiac Surgery, National Institute of Cardiovascular Diseases, Dhaka, BGD; 2 Dermatology, Bangladesh Medical University (BMU), Dhaka, BGD; 3 Cardiac Surgery, Bangladesh Medical University (BMU), Dhaka, BGD

**Keywords:** cabg, coronary artery bypass, diabetes mellitus, glargine, glycaemic control, insulin

## Abstract

Background: Hyperglycemia is common after coronary artery bypass grafting (CABG) among patients with diabetes and has been associated with increased morbidity rates. The conventional strategies involving sliding-scale insulin therapy alone or continuous IV infusion alone are frequently ineffective due to the lack of basal insulin support.

Objectives: To evaluate whether adding subcutaneous glargine to continuous intravenous regular insulin enhances glycaemic control and postoperative outcomes among diabetic patients who undergo CABG.

Methods: This is a prospective observational study carried out among 260 diabetic patients scheduled to undergo elective CABG at the National Institute of Cardiovascular Diseases, Dhaka. The patients were randomly divided into two groups: Group A received continuous IV regular insulin alone, and Group B received continuous IV regular insulin and once-daily subcutaneous glargine. Blood glucose levels were recorded until the second day after the operation. The postoperative complications and outcomes were recorded.

Results: The mean postoperative blood glucose levels were significantly lower in Group B compared to Group A (mean 9.25 ± 0.84 vs. 12.14 ± 0.77 mmol/L; p <0.001). Group B also had a significantly lower incidence of acute kidney injury (2.3% vs. 23.1%, p <0.001) and sternal wound infection (0.8% vs. 13.1%, p <0.001). The duration of postoperative hospitalisation was less for Group B (9.5 ± 1.3 vs 10.5 ± 1.7 days, p <0.001). Mortality rates were equally matched by the two groups.

Conclusion: The study concludes that combining subcutaneous glargine with continuous intravenous infusion of insulin achieves optimal glycaemic control following surgery and reduces morbidity amongst diabetic CABG patients when compared with intravenous insulin alone.

## Introduction

Diabetic patients have more progressive and diffuse coronary stenosis and more end-organ dysfunction, including renal insufficiency and neurological deficits [1,2]. The prevalence of diabetes in patients requiring coronary artery bypass graft (CABG) surgery continues to increase, and it is now estimated that almost 20-30% of CABG patients will have DM or metabolic syndrome. Furthermore, a repeat revascularisation procedure might be needed for diabetic CABG patients, including a 24% higher risk of readmission due to cardiac-related problems and a 44% higher risk for re-hospitalisation for any other cause [3].

Patients with diabetes generally have worse outcomes after acute MI and left ventricular dysfunction than non-diabetic patients and are more likely to have perioperative mortality and reduced long-term survival after CABG surgery. These results were thought to be irreversible due to impaired endothelial function, more diffuse coronary disease, and abnormal fibrinolytic and platelet function, which are more commonly seen in patients with diabetes mellitus, resulting in lower graft patency [4-6].

The blood vessel endothelium is more adversely affected by fluctuations in blood sugar levels than by persistent chronic hyperglycaemia. Recent studies have shown that acute and chronic fluctuations in blood sugar can increase oxidative stress in diabetic patients, leading to cellular dysfunction and tissue damage [7,8]. Therefore, stable and optimum glycaemic control is vital for better early outcomes following coronary artery bypass graft surgery.

In the case of tight glycaemic control by continuous intravenous insulin infusion, there is a potential for hypoglycaemia in the postoperative period when the patient is still in the compensatory phase of recovering from surgical stress, which is very important as it can cause irreversible brain damage [9]. Moreover, rapid changes in glucose levels are also dangerous and harmful, and, again, difficult to ascertain when testing for those patients with intermittent glucose levels. This situation is further complicated by the fact that patients after cardiac surgery in the intensive care unit are often sedated for hours or even days after surgery and are unlikely to show symptoms of hypo- or hyperglycaemia [10,11].

In a landmark study, Malmberg and coworkers first demonstrated that maintaining serum glucose levels less than 11.1 mmol/L in diabetic patients with acute myocardial infarction (MI) using a continuous insulin infusion reduced mortality by 30% [12]. Van den Berghe and coworkers, in a study involving 1548 ventilated patients admitted to a surgical ICU, of which 62% had undergone cardiac surgery, found that mortality was significantly reduced in those patients requiring three days or more of ICU care if serum glucose level was maintained between 4.4 and 6.1 mmol/L [13].

In the past, the standard for the treatment of hyperglycaemia in hospitalised patients was using a regular insulin regimen on a sliding scale. However, the sliding scale regular insulin approach is widely acknowledged as ineffective. Yeldandi and coworkers have shown that the use of basal and prandial insulin better corrects hyperglycaemia in hospitalised patients and improves blood glucose control compared to sliding-scale regular insulin [14].

Lazar and coworkers found results similar to those of Furnary and coworkers, who demonstrated that maintaining serum glucose levels between 5.6 and 8.3 mmol/L using continuous insulin infusions decreased the incidence of deep sternal infections, lowered mortality, improved ventricular function, decreased the need for inotropic support, and shortened hospital stay [15-18]. Based on these observations, the Society of Thoracic Surgeons Work Force on Evidence-Based Surgery has recommended continuous insulin infusions to keep serum glucose levels less than 11.1 mmol/L in all patients during cardiac surgery and their stay in the intensive care unit (ICU).

A pharmacokinetic profile for a long-acting insulin analogue (glargine; Lantus, SoloSTAR®Pen) shows its effect two hours after administration, and its effect lasts for around 24 hours without a peak effect [19]. It is expected that basal insulin-like glargine administration once daily causes a significant reduction not solely in blood glucose levels (without inflicting hypoglycaemia) but also in the daily requirement of human insulin infusion. The combination of glargine and continuous insulin infusion in patients undergoing CABG prevents fluctuation in blood glucose levels and provides better glycaemic control, in addition to decreasing postoperative morbidity [20,21]. There is limited evidence from low-resource regions on the role of basal insulin in CABG patients.

This study aimed to compare glycaemic control and early outcomes between patients receiving continuous intravenous insulin alone versus intravenous insulin plus subcutaneous glargine following CABG.

## Materials and methods

Study design and population, sampling, and intervention protocol

In a previous prospective observational study, data were collected from diabetic patients who fulfilled the inclusion criteria (all elective CABG patients). However, exclusion criteria (s/p cardiac surgery or revascularisation, i.e., CABG/valve replacement/ICR, conversion to CPB, prior MI within six weeks) were applied for those admitted patients to the department of Cardiac Surgery, National Institute of Cardiovascular Diseases (NICVD), Sher-E-Bangla Nagar, Dhaka-1207 for coronary artery bypass graft (CABG) surgery. A total of 260 patients undergoing coronary artery bypass grafting were divided into the control (Group A - diabetic patients whose blood glucose was controlled by continuous regular insulin without subcutaneous glargine) or study (Group B - diabetic patients whose blood glucose was controlled by continuous regular insulin with subcutaneous glargine) group. Subcutaneous glargine (injection Lantus) was started at 12 international units, whereas continuous intravenous regular insulin (CII) was given in the infusion pump just after moving the patient to the ICU. For CII, regular insulin was prepared by diluting 50 units in 50 ml of 0.9% normal saline. The initial insulin infusion rate was set at 1 U/h. Blood glucose (BG) was measured hourly. The infusion rate was titrated according to a protocol: if BG > 8 mmol/L, the rate was increased by 1 U/h for every 5 mmol/L above target; if BG was within target (8 mmol/L), the rate was maintained; if BG < 4.5 mmol/L, the infusion was decreased or stopped [16]. Blood sugar levels were recorded in both groups in the ICU up to the second postoperative day. An AcuChec glucose analyser machine for finger strip examination and a Beckman Coulter AU 480 analyser for lab investigation were used for glucose estimation. For data analysis, average daily blood glucose levels were calculated from all blood glucose samples obtained from the day of operation up to the second POD. Each patient was also evaluated by continuous ECG monitoring up to 7 AM of the second POD & daily 12-lead ECG at least up to the second POD. The evaluation was extended if needed.

Serum potassium levels were maintained between 4.0 and 5.0 mmol/L through the administration of intravenous potassium. In the ICU, this was done according to a standardised protocol. Oral potassium supplementation was given to maintain these levels once patients tolerated enteral nutrition. Furthermore, AKI was measured by KDIGO AKI staging. 

The study was approved by the Institutional Review Board of NICVD (Reference: Nicvd/Academic/Ethical/2021/3040; Date: 21.08.2021). Written informed consent was obtained from all participants. A detailed history, predisposing factors, and demographic profile were taken using a predesigned questionnaire and recorded in a data collection sheet. The collected information was entered into Microsoft Excel and IBM Corp. Released 2020. IBM SPSS Statistics for Windows, Version 26. Armonk, NY: IBM Corp. Collected all questionnaires and checked carefully to identify errors in collecting data. Data processing work consisted of registration of schedules, editing, coding, and computerisation, preparation of dummy tables, analysis, and matching data. The technical matter of editing, encoding, and computerisation was looked at by the researcher.

Data analysis

Continuous variables were expressed as mean ± standard deviation (SD) and compared using the independent Student’s t-test (two-tailed, assuming equal variances). Categorical variables were expressed as frequencies and percentages and compared using the chi-square test; Fisher’s exact test was applied when expected cell counts were <5. All statistical tests were two-tailed and were compared using the chi-square test or Fisher’s exact test when appropriate. All statistical analyses were performed using IBM Corp. Released 2020. IBM SPSS Statistics for Windows, Version 26. Armonk, NY: IBM Corp. A p-value < 0.05 was considered statistically significant. Exact test statistics (t, χ²) and degrees of freedom are reported alongside p-values in the figure and table legends.

## Results

The clinical characteristics of patients are shown in Table 1. No significant difference was found concerning age, sex, or BMI regarding history in terms of preoperative RBS, eGFR, and LVEF between the two groups, but males are predominant in both groups. Preoperatively, diabetes control was achieved either by insulin, oral hypoglycemic agents, or diet. However, these differences were not statistically significant between the two groups.

**Table 1:** Clinical characteristics of patients (n=260) Data are expressed as mean±*SD or number (percentage). An independent samples t-test was used for continuous variables (age, RBS, HbA1c), and Pearson's chi-square (χ²) test was used for categorical variables. For age: t(258) = -0.285, p = 0.777; for gender: χ²(1, N=260) = 0.098, p = 0.754; for BMI: χ²(3, N=260) = 0.599, p = 0.891; for smoking: χ²(1, N=260) = 2.405, p = 0.121; for hypertension: χ²(1, N=260) = 0.067, p = 0.796; for hyperlipidaemia: χ²(1, N=260) = 0.278, p = 0.598; for H/O MI: χ²(1, N=260) = 0.602, p = 0.438; for multiple comorbidities: χ²(1, N=260) = 0.221, p = 0.638; for preoperative diabetes treatment: χ²(2, N=260) = 1.270, p = 0.530; for RBS: t(258) = 0.826, p = 0.410; for HbA1c: t(258) = -0.766, p = 0.444; for eGFR: χ²(1, N=260) = 0.800, p = 0.371; for LVEF: χ²(3, N=260) = 0.319, p = 0.953. A p-value < 0.05 was considered statistically significant. = Significant; ns = non-significant.*

Variables		Group A (n_1_=130)	Group B (n_2_=130)	p-value
Age (year) (mean ± SD)		51.57±9.49	52.23±8.67	0.777^ns^
Gender (%)	Male	99 (76.15%)	104 (80.00%)	0.754^ns^
	Female	31 (23.85%)	26 (20.00%)	
BMI	Underweight (<18.5)	0 (0.00%)	0 (0.00%)	0.891^ns^
	Normal (18.5-24.9)	73 (56.15%)	70 (53.85%)	
	Overweight (25.0-29.9)	47 (36.15%)	47 (36.15%)	
	Obese (>30.0)	10 (7.69%)	13 (10.00%)	
Smoking (%)		52 (40.00%)	64 (49.00%)	0.121^ns^
Hypertension (%)		65 (50.00%)	70 (53.85%)	0.796^ns^
Hyperlipidaemia (%)		67 (51.54%)	73 (56.15%)	0.598^ns^
H/O Myocardial Infarction (%)		98 (75.38%)	99 (76.15%)	0.438^ns^
Multiple comorbidities (%)		31 (23.85%)	47 (36.15%)	0.638^ns^
Preoperative modalities of diabetes treatment	Diet (%)	13 (10.00%)	13 (10.00%)	0.530^ns^
	Oral hypoglycemic agent (%)	82 (63.08%)	65 (50.00%)	
	Insulin (%)	35 (26.92%)	52 (40.00%)	
RBS (mmol/L) (mean ± SD)		10.16±2.75	9.55±2.89	0.410^ns^
HbA_1_C (%) (mean ± SD)		7.62±1.01	7.86±1.37	0.444^ns^
eGFR (ml/min/1.73 m^2^)	Normal (>90)	104 (80.00%)	91 (70.00%)	
	Early Kidney Disease (60-90)	26 (20.00%)	39 (30.00%)	0.371^ns^
LVEF (%)	Normal (>55%)	61 (46.92%)	56 (43.07%)	0.953^ns^
	Mild (45-55%)	35 (26.92%)	47 (36.15%)	
	Moderate (35-45%)	21 (16.15%)	17 (13.08%)	
	Severe (<35%)	13 (10.00%)	10 (7.69%)	

Preoperatively, the type of surgery, duration of surgery (hours), total number of grafts, blood glucose, preoperative MI, and arrhythmia were not significantly different between the two groups, as shown in Table 2.

**Table 2:** Comparison of peroperative variables (n=260) Independent samples t-test was used for continuous variables (duration of surgery) and Pearson Chi-square (χ²) test was used for categorical variables. For type of surgery: χ² (1, N=260) = 0.098, p = 0.754; for duration of surgery: t (258) = -1.772, p = 0.078; for total number of grafts: χ² (3, N=260) = 3.861, p = 0.269; for per-operative MI: Fisher's Exact test, p = 1.000; for per-operative arrhythmia: Fisher's exact test, p = 1.000. A p-value < 0.05 was considered statistically significant. ns= not significant. *

Variables		Group A (n_1_=130)	Group B (n_2_=130)	p-value
Type of Surgery (%)	OPCAB	99 (76.15%)	104 (80.00%)	0.754^ns^
	CCAB	31 (23.85%)	26 (20.00%)	
Duration of Surgery (Hours) (mean ± SD)		4.34±0.58	4.67±0.81	0.078^ns^
Total Number of Grafts (%)	Arterial (1)	9 (6.67%)	8 (6.15%)	0.269^ns^
	Arterial (1), Venous (1)	52 (40.00%)	47(36.15%)	
	Arterial (1), Venous (2)	52 (40.00%)	61 (46.93%)	
	Arterial (1), Venous (3)	17 (13.33%)	14 (10.76%)	
	Others	0 (0%)	0 (0%)	
Per-operative MI (%)		4 (3.08%)	3 (2.31%)	1.000^ns^
Per-operative Arrhythmia		9 (6.67%)	10 (7.69%)	1.000^ns^

Table 3 shows that postoperative blood glucose on the day of operation, at first POD, at second POD, and at third POD in the ICU, including average blood glucose level up to second POD, was significantly higher in Group A compared to Group B.

**Table 3:** Comparison of postoperative blood glucose level (up to second POD) (n=260) Mean blood glucose levels (±SD) were compared between patients receiving continuous IV insulin only (Group A, n=130) and those receiving IV insulin plus subcutaneous glargine (Group B, n=130). Comparisons at each time point were performed using independent Student’s t-tests (two-tailed). Operation day: t (258) =8.66, p<0.001; POD1: t (258) =23.10, p<0.001; POD2: t (258) =30.36, p<0.001. A p-value < 0.05 was considered statistically significant. * = Significant; ns = non-significant. *

Postoperative Variables (Average)	Group A (n_1_=130) Mean ± SD	Group B (n_2_=130) Mean ± SD	p-value
Blood glucose on the day of operation (mmol/L)	12.15±1.45	10.66±1.32	<0.001*
Blood glucose at 1^st^ POD (mmol/L)	12.25±1.35	9.04±0.83	<0.001*
Blood glucose at 2^nd^ POD (mmol/L)	12.05±1.30	8.04±0.76	<0.001*
Average (per day) postoperative blood glucose level (mmol/L)	12.14±0.77	9.25±0.84	<0.001*

Table 4 shows that the incidence of postoperative sternal wound infection was 17 (13.08%) in Group A and 1 (0.77%) in Group B, and acute kidney injury was 30 (23.08%) in Group A and 3 (2.31%) in Group B. Sternal wound infection and acute kidney injury were significantly higher in Group A. Postoperative hospital stay was significantly lower in Group B compared to Group A. The mortality rate, along with other postoperative morbidity, was not significantly different between the two groups.

**Table 4:** Distribution of the study patients by postoperative outcome (n=260) Independent samples t-test was used for continuous variables (duration of ICU stay, postoperative hospital stays), and Pearson's chi-square (χ²) or Fisher's exact test was used for categorical variables. For mechanical ventilation: χ²(1, N=260) = 0.301, p = 0.584; for duration of ICU stay: t(258) = 1.342, p = 0.182; for postoperative hospital stays: t(258) = 5.36, p=1.84×10⁻⁷; for in-hospital mortality: Fisher's Exact Test, p = 1.000; for neurological complication: Fisher's Exact Test, p = 1.000; for pulmonary complication: Fisher's Exact Test, p = 1.000; for acute kidney injury: χ²(1, N=260) = 25.30, p=4.90×10⁻⁷; for sternal wound infection: Fisher's Exact Test, p = 9.27×10⁻⁵. A p-value < 0.05 was considered statistically significant. Significant, ns= not significant.*

Postoperative outcome	Group A (n_1_=130)	Group B (n_2_=130)	p-value
Mechanical Ventilation (>6 hours)	82 (61.54%)	91 (70.00%)	0.584^ns^
New Arrhythmia (%)	0 (0%)	0 (0%)	0
Post-operative MI (%)	0 (0%)	0 (0%)	0
Duration of ICU Stay (days) (mean ± SD)	5.80±0.71	5.43±1.31	0.182^ns^
Postoperative hospital stays (days) (mean ± SD)	10.53±1.66	9.53±1.33	<0.001*
In-hospital mortality (%)	3 (2.31%)	3 (2.31%)	1.000^ns^
Neurological Complication (%)	9 (6.67%)	9 (6.67%)	1.000^ns^
Pulmonary Complication (%)	3 (2.31%)	3 (2.31%)	1.000^ns^
Acute Kidney Injury (%)	30 (23.08%)	3 (2.31%)	<0.001*
Sternal Wound infection (%)	17 (13.08%)	1 (0.77%)	<0.001*

## Discussion

This study was conducted at NICVD and included a statistically adequate number of 260 diabetic patients. In those patients, subcutaneous glargine injections in addition to continuous intravenous insulin infusion were used in group B. This is the first study from Bangladesh evaluating glargine with continuous intravenous short-acting insulin infusion in CABG patients.

In this study, the age of the patients ranged from 35 to 70 years, with a mean of 51.57±9.49 years in group A and 52.23±8.67 years in group B. Patients of Group A are younger than those of Group B, but the difference is statistically not significant, a reflection of the age of patients has less chance to influence the outcome of the study, and this is consistent with a study by Forouzannia and coworkers [20].

In this study, most of the patients were male in both groups, A and B, and were 76.15% and 80.00%, respectively. The difference between the groups is not statistically significant. This figure is consistent with a study conducted by Gandhi and coworkers [3].

In this study, the normal body mass index (BMI) of the patients was higher in group A (56.15%) than in group B (53.85%), but the difference is not statistically significant. In addition, hypertension and hyperlipidaemia in patients of groups A and B were 50.00%, 51.54%, 53.85%, and 56.15%, respectively. The difference between the groups is not statistically significant. These findings are consistent with the study of Forouzannia and coworkers, whereas the history of smoking and MI in group A (40.00%, 56.67%) and group B (60.00%, 46.67%), respectively, were inconsistent with this study [20]. However, the difference among the groups is not statistically significant.

In this study, preoperative investigation findings in terms of RBS, HbA1C, LVEF, and eGFR were not significantly different among the groups. These findings are consistent with the studies of Forouzannia and coworkers [20] and Gandhi and coworkers [3].

In this study, oral hypoglycaemic agents were the most common antidiabetics used preoperatively by the patients, as they were used in group A (63.08%) and group B (50.00%). In group A, 26.92% and in group B, 40% received insulin. The differences among the groups are statistically insignificant and also consistent with the study conducted by Forouzannia and coworkers [20].

In this research study, the duration of surgery is longer in group B than in group A, with a mean of 4.34±0.58 hours and 4.67±0.81 hours, respectively, but the difference is statistically insignificant, and the number of grafts was nearly similar between groups A and B. The preoperative mean blood glucose level was 165.63±20.71 mg/dl and 145.61±23.85 mg/dl in groups A and B, respectively. Although blood glucose levels were lower in group B, the difference was not statistically significant. The perioperative findings of this study in terms of the type of surgery, duration of surgery, the total number of grafts, the occurrence of MI, and arrhythmia were not statistically significant between group A and group B. These findings are similar to the study of Forouzannia and coworkers [20].

Furthermore, this study shows that overall glycaemic control was significantly better in group B. Postoperative mean blood glucose levels on the day of operation, first POD, and second POD, including three-day average blood glucose levels, were lower in group B (10.66±1.32; 9.04±0.83; 8.04±0.76; 9.25±0.84, respectively) than in group A (12.15±1.45; 12.25±1.35; 12.05±1.30; 12.14±0.77, respectively), and the differences are statistically significant. These findings are similar to the studies of Forouzannia and coworkers and Gandhi and coworkers [3,20].

Further, in this study, the occurrence of postoperative acute kidney injury was 23.08% in Group A and 2.31% in Group B. However, the occurrence of postoperative sternal wound infection was noticeable between Group A and Group B, which was 13.08% in Group A. The mean postoperative hospital stay was 10.53±1.66 days and 9.53±1.33 days in Group A and Group B, respectively. Postoperative hospital stays, acute kidney injury, and sternal wound infection were statistically significant and are consistent with the study conducted by Gandhi and coworkers [3]. Duration of ICU stay was 5.80±0.71 days in Group A and 5.43±1.31 days in Group B. Three (2.31%) patients died postoperatively in the hospital in both groups, but the difference is not statistically significant. In addition, mechanical ventilation duration (>6 hours) and pulmonary and neurological complications were 61.54%, 2.31%, and 6.67%, respectively, in group A, whereas 70.00%, 2.31%, and 6.67% were in group B. The difference among the groups is not statistically significant. This figure is consistent with a study conducted by Gandhi and coworkers [3].

Though the first study from Bangladesh evaluating basal insulin in CABG, our study has some limitations because it has a relatively small sample of patients for a limited time. Nevertheless, the study was conducted in a single centre, and multiple surgical teams performed the procedures. Propensity scoring of the collected data was not done. We anticipate that a large sample can be used for a long period to make useful guidelines for postoperative insulin use. And the study can be conducted in multiple cardiac centres to determine standard diabetic control techniques. However, standard management of perioperative hyperglycaemia can be used to maintain safe and successful blood glucose control following off-pump CABG in diabetic patients. For this, proper insulin administration during the immediate postoperative period is necessary to keep blood glucose levels as low as possible.

## Conclusions

In Bangladesh, coronary artery bypass grafting is becoming more common, and off-pump coronary artery bypass surgery is becoming ever more popular. Among the different risk factors, diabetes mellitus (DM) is a significant risk factor for a patient undergoing off-pump coronary artery bypass surgery and having poor postoperative results. In addition, in diabetic patients, hyperglycaemia in the initial postoperative period is a significant risk factor for sternal wound infection, new-onset arrhythmia, longer ICU and hospital stays, neurological complications, pulmonary complications, sternal wound infection, and increased death.

As a result, diabetes should be managed as effectively as possible. Our study suggests that adding subcutaneous glargine to continuous insulin infusion provides better postoperative glycaemic control and reduces morbidity in diabetic CABG patients.
